# Hybrid stepwise external wrapping for type a acute aortic dissection with cerebral Malperfusion

**DOI:** 10.1186/s13019-020-01381-y

**Published:** 2021-01-11

**Authors:** Yoshihiro Suematsu, Satoshi Nishi, Daisuke Arima, Akihiro Yoshimoto

**Affiliations:** Department of Cardiovascular Surgery, Tsukuba Memorial Hospital, 1187-299 Kaname, Tsukuba, Ibaraki, 300-2622 Japan

**Keywords:** Type a acute aortic dissection, Stepwise external wrapping, Cerebral Malperfusion

## Abstract

**Background:**

Acute aortic dissection (AAD) is a life-threatening condition which can lead to coronary, brachiocephalic or branch vessel malperfusion, as well as aortic valve insufficiency, or aortic rupture. Mortality of surgical treatment in high-risk or elderly patients with Type A AAD (TAAAD) still remains high, and treatment for such patients remains controversial. We report a successful treatment of TAAAD with a communicating false lumen in a 60-year-old man with acute hemi-cerebral malperfusion.

**Case presentation:**

The ascending aorta was wrapped with stepwise external wrapping (SEW) procedure, and subsequent thoracic endovascular aortic repair (TEVAR) was successfully performed. The patient was discharged in good physical condition without any complications**.**

**Conclusions:**

Hybrid therapy with SEW and TEVAR with TAAAD associated with major cerebral malperfusion should be considered, especially in patients for whom open surgery is extremely risky.

## Introduction

We herein report a successful less-invasive hybrid therapy of type A acute aortic dissection (TAAAD) in a 60-year-old man with acute hemi-cerebral malperfusion.

## Case presentation

**Fig. 1:**
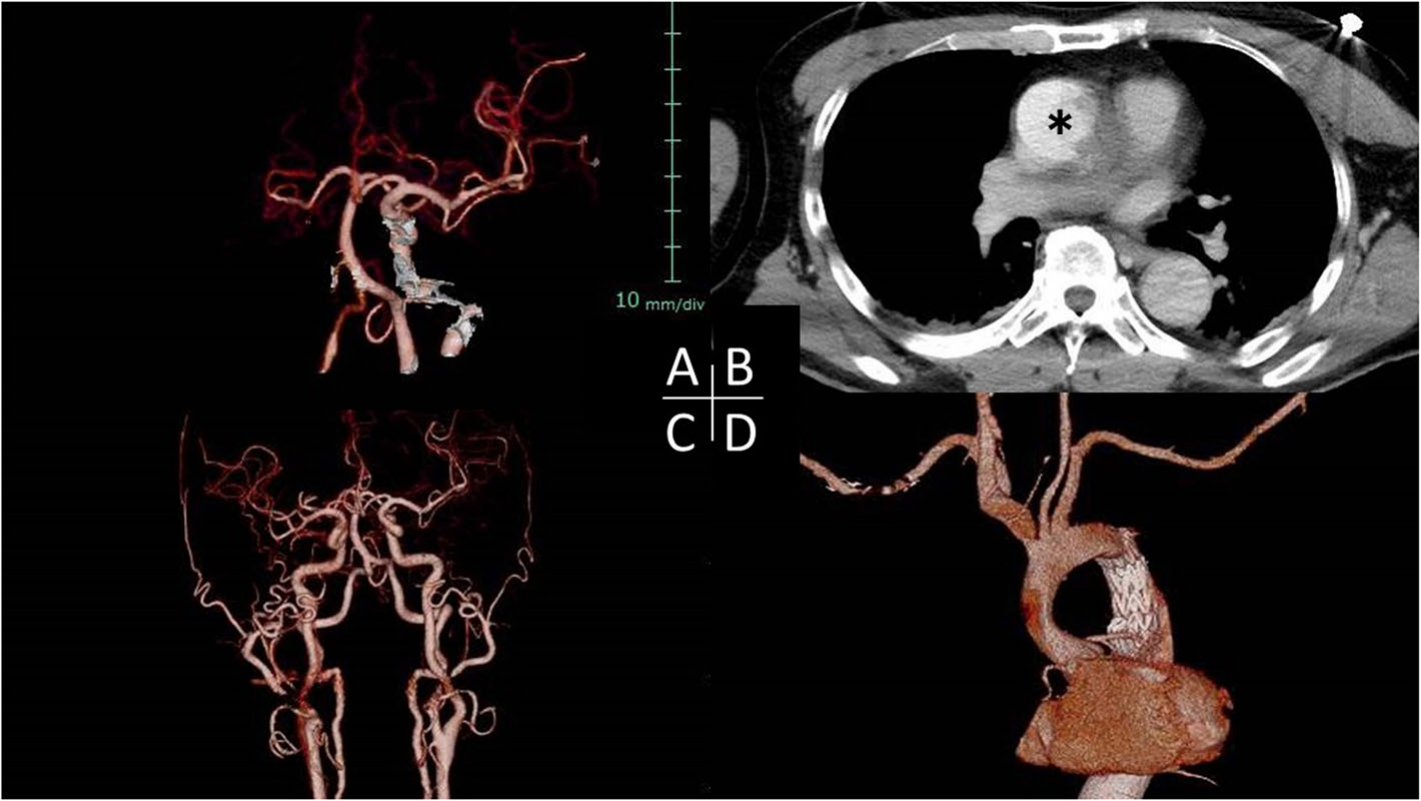
**a** Preoperative three-dimensional reconstruction images of computed tomography (3-D CT images), demonstrating non-visualization of the right cerebral artery. **b** Transverse-view image showing the collapsed true lumen in the ascending aorta. Asterisk showing the false lumen. **c** Postoperative3-D CT image after the stepwise external wrapping procedure showing normal cerebral arteries. **d** Postoperative 3-D CT image showing successful thoracic endovascular stent-graft deployment into the thoracic aorta without any endoleak, and patent neck vessels

The patient was a 60-year-old man with a medical history of untreated severe sleep apnea syndrome, hypertension, and paroxysmal atrial fibrillation without any medication because of low compliance. At early morning, he had sudden back pain and was unresponsive. He was found in his car, and emergently transported to a tertiary medical facility. He demonstrated left hemiplegia, conjugate deviation of the eyes, and convulsions. He was intubated and computed tomography (CT) showed absence of perfusion to right whole cerebral artery without any irreversible brain damage and TAAAD with a 20 mm of intimal tear in the descending aorta, and obvious cardiac tamponade (Fig. 1a-d). The aortic dissection was extended to the just distal of the Vasalva sinus. Four cardiac centers in tertiary hospitals refused to accept him due to inoperability because of probable brain damage. He was finally transferred to our hospital. At arrival, 5 h had already passed from the onset of his condition, and he still showed deep coma and shock vitals with inotropic support. The patient’s estimated standard European System for Cardiac Operative Risk Evaluation (EuroSCORE) II result was 59.25. His family strongly wished to have surgical intervention.

**Fig. 2:**
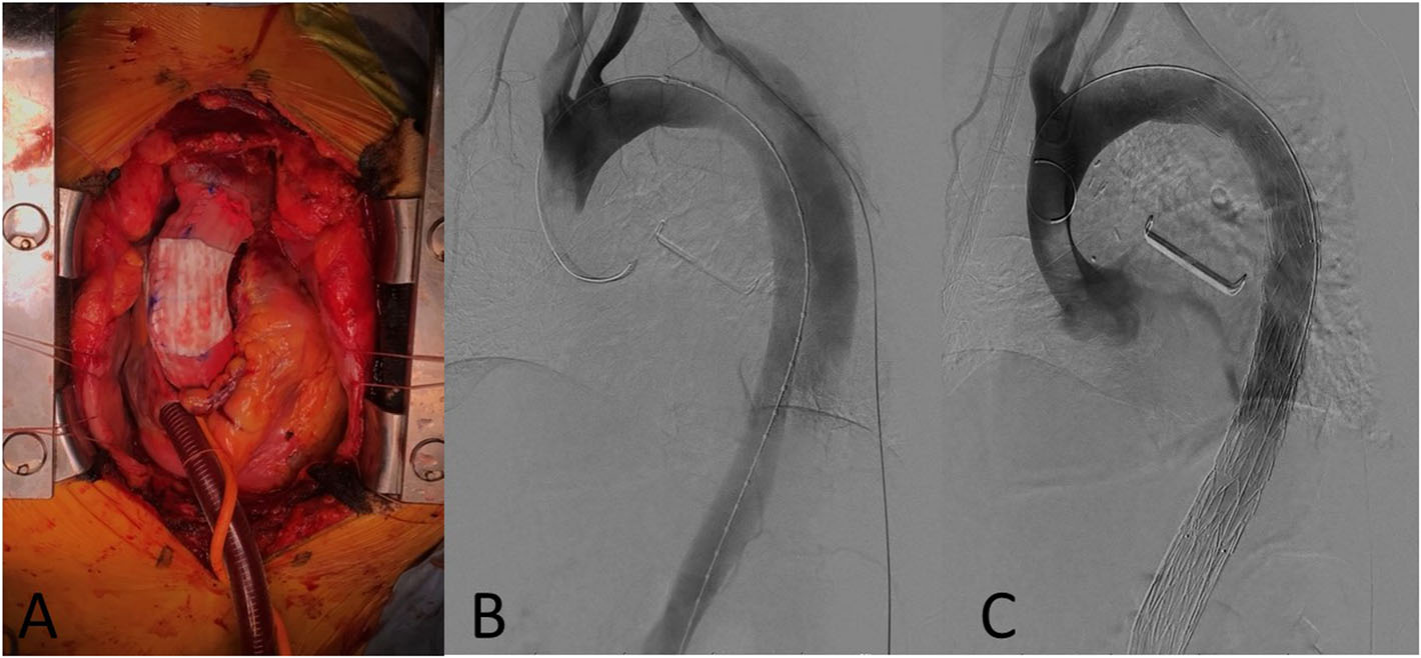
**a** The operative images. Three pieces of an artificial graft tailored together, and approximated so as to tightly wrap the ascending aorta. **b** Digital Subtraction Angiography (DSA) before the thoracic endovascular stent-graft deployment. **c** DSA after the thoracic endovascular stent-graft deployment, showing no endoleak

We performed emergency surgery. Initially, median sternotomy was performed, and cardiopulmonary bypass was established through femoral arterial cannulation and single two-stage venous cannulation via right atrial appendage. Intraoperative transesophageal echocardiography demonstrated mild aortic regurgitation. We then proceeded to treat the patient using the new surgical approach devised by us for high-risk patients, reported previously (stepwise external wrapping: SEW) [1, 2]. The ascending aorta was carefully separated from the pulmonary arterial trunk and right pulmonary artery. Pieces of a Triplex artificial graft® (Vascutek Terumo, Tokyo, Japan) were tailored and placed around the aorta from the coronary ostia to the innominate artery and approximated so as to tightly wrap the ascending aorta (Fig. 2a). At this moment, near-infrared spectroscopy cerebral oximetry with Invos™ (Medtronic®, Minneapolis, MN, USA) revealed significant improvement of the cerebral perfusion. The operation time was 88 min and cardiopulmonary bypass time was 29 min. 3 h later, he was extubated without any neurological damage. One week later, thoracic endovascular aortic repair (TEVAR) was performed. The intimal tear was located in the descending aorta 104 mm distal from the left subclavian artery (Zone T5). Since the distance from the proximal landing zone (Zone 3) was sufficient, we placed A 10% oversized aortic stent graft proximally 50 mm from the entry and distally 150 mm from the entry with Navion® (22–22-180; Medtronic, Santa Rosa, CA, USA) and TX-D extension® (24–24-80; Medtronic). In addition, we placed a TX-D® (barestent; 36–123; Medtronic) and TX-D® (barestent; 36–164; Medtronic). Finally, distal end was located just above the aortic bifurcation.

The length of hospitalization was 14 days. No neurological complications or minor complications were encountered. Postoperative CT showed that proper positioning of the stent graft without any endoleak, good brain perfusion (Fig. 1c, d). The patient was discharged in good physical condition without any complications and is doing well at 6 months after surgery.

## Comments

To the best of our knowledge, this is the first reported case in the literature of successful “hybrid” therapy with SEW and TEVAR for a TAAAD associated with major cerebral malperfusion.

The application of TEVAR has dramatically changed the treatment paradigm for disease of the descending thoracic aorta. It has been shown to be valid rescue option for patients with acute aortic dissection who are not eligible for open surgical repair. The first successful report of TEVAR in a patient with acute aortic dissection was published by Dorros et al. in 2000 [3]. Subsequently, several other investigators have reported successful treatment of TAAAD using TEVAR [4, 5]. However, TEVAR for TAAAD remains challenging because of the anatomical vicinity of the ascending aorta to the aortic valve, coronary artery, and brachiocephalic artery. In the current case, TEVAR alone for the ascending or descending aorta would have been inadequate, because the aortic dissection extended to the Valsalva sinus, and the patient had massive cardiac tamponade. Our SEW procedure was useful for temporarily controlling the ascending aortic rupture, which is usually difficult to accomplish by total TEVAR with debranching or fenestration, and for preventing future aneurysmal change of the ascending aorta.

In our patient reported herein, the initial SEW alone relieved the cerebral malperfusion, presumably because of the decrease in blood flow from the true lumen into the false lumen. Since the cardiac output remained unchanged, the blood flow in the true lumen increased in spite of the decreased blood flow in the false lumen, which led to dramatic improvement of the blood flow into the right innominate artery. Intraoperative trans-esophageal echocardiography (TEE) at the level of the descending aorta also showed a change in the balance of the flow between the true lumen and false lumen.

In selected extremely high-risk patients, like in this case, which would generally have been considered to be inoperable, hybrid therapy with SEW and TEVAR may be a viable alternative to therapeutic abstention or conventional surgery.

## Conclusions

Hybrid therapy with SEW and TEVAR with TAAAD associated with major cerebral malperfusion should be considered, especially in patients for whom open surgery is extremely risky.


**Additional file 1: Video S1.** Stepwise External wrapping procedure. A median sternotomy was made and cardiopulmonary bypass was established through femoral arterial cannulation and single two-stage venous cannulation via right atrial appendage. The ascending aorta was carefully separated from the main pulmonary arterial trunk and right pulmonary artery. Utmost care was taken during the dissection to avoid tearing the dissected aorta. Three pieces of an artificial graft were tailored, placed around the aorta from the coronary ostia up to the innominate artery, in a stepwise fashion, and approximated with a running suture so as to tightly wrap the ascending aorta. Then, Bioglue® (CryoLife Inc., Kennesaw, GA, USA) was applied around the graft to prevent oozing-type bleeding through the grafts and early graft migration.

## Data Availability

Yes

## Abbreviations

AAD: Acute aortic dissection
TAAAD: Type A Acute aortic dissection
SEW: Stepwise external wrapping
CT: Computed tomography
TEE: Trans esophageal echocardiography
TEVAR: Thoracic endovascular aortic repair
